# Novel human single-domain antibodies exert potent anti-tumor activity by targeting EGF-like repeat epitope of EpCAM

**DOI:** 10.3389/fphar.2025.1530268

**Published:** 2025-02-13

**Authors:** Xiaofeng Zhou, Zhifang Liu, Weixiong Zhang, Lin Dai, Tao Chen, Zexiong Lin, Hong Pan, Qi Qi, Henry Wei

**Affiliations:** ^1^ State Key Laboratory of Bioactive Molecules and Druggability Assessment, MOE Key Laboratory of Tumor Molecular Biology, Guangdong Province Key Laboratory of Pharmacodynamic Constituents of TCM and New Drugs Research, Department of Pharmacology, School of Medicine, Jinan University, Guangzhou, China; ^2^ Department of Cell Biology and Institute of Biomedicine, National Engineering Research Center of Genetic Medicine, Guangdong Provincial Key Laboratory of Bioengineering Medicine, College of Life Science and Technology, Jinan University, Guangzhou, Guangdong, China; ^3^ Department of Ophthalmology, Shandong Provincial Hospital Affiliated to Shandong First Medical University, Jinan, Shandong, China

**Keywords:** EpCAM, antibody, single-domain antibody, cancer stem cells, cancer therapy

## Abstract

**Introduction:**

EpCAM (Epithelial cell adhesion molecule) is a key cancer stem cell marker involved in cancer progression, making it an important target for both diagnosis and therapy. Despite efforts using anti-EpCAM monoclonal antibodies (mAbs), their anti-tumor effects have been limited. Single-domain antibodies (sdAbs), in contrast, offer advantages such as efficient tumor penetration and reduced immunogenicity. This study aims to screen and explore novel sdAbs targeting EpCAM for cancer therapy.

**Methods:**

A critical EGF-like repeat epitope on the EpCAM extracellular domain was selected for screening a human sdAb library via phage display. The selected sdAbs were purified and their anti-cancer activity was validated through specific binding with the EpCAM peptide. The effects of these sdAbs on cell proliferation, migration, invasion, and apoptosis were tested *in vitro*, and their anti-tumor activity was assessed in a xenograft model.

**Results:**

Five fully human anti-EpCAM sdAbs were isolated, all of which specifically bound to the EpCAM peptide and showed selective binding to various cancer cell lines, but not to 293T and 3T3 cells. Functional assays demonstrated that these sdAbs significantly inhibited cancer cell proliferation, migration, and invasion, and induced apoptosis. Notably, two sdAbs (aEP3D4 and aEP4G2) exhibited potent anti-tumor effects *in vivo*, significantly reducing tumor volume and weight in a mouse xenograft model.

**Discussion:**

This study provides compelling evidence that targeting EpCAM with sdAbs is a promising approach for cancer treatment. The identified anti-EpCAM sdAbs exhibit substantial anti-tumor activity both *in vitro* and *in vivo*, suggesting they are strong candidates for future therapeutic applications in cancer therapy.

## Introduction

Epithelial cell adhesion molecule (EpCAM) is a cell adhesion protein mediating calcium-independent homophilic cell-cell adhesion. It is composed of a long extracellular domain, a transmembrane domain and a short intracellular domain (Yahyazadeh Mashhadi et al., 2019). Proteolysis of EpCAM transmembrane domain results in shedding of its extracellular domain and accumulation of its intracellular domain to nucleus, which triggers oncogenic signaling pathway and epithelial tumor malignancy (Carpenter and Brewer, 2009; Wang and Zoller, 2019). The EpCAM intracellular domain is increased in cancer cells of breast, prostate, head and neck and esophagus compared to their corresponding normal tissues with EpCAM cell membrane localization (Wang and Zoller, 2019). EpCAM is overexpressed in many human cancers. Its over-expression involves in cancer cell proliferation, invasion, metastasis, malignant potential and therapy resistance (Kalantari et al., 2022; Hwang et al., 2022). EpCAM overexpression was found in 98% and 100% cancer cells respectively in metastatic stage of esophageal squamous cell carcinoma (ESCC) and esophageal adenocarcinoma (EACA) and associated with poor prognosis in ESCC patients (Krishnamurthy and Jimeno, 2018; Ahamadi-Fesharaki et al., 2019). In prostate cancer cells, EpCAM has tumor initiation potential and is involved in proliferation, invasion, metastasis and chemo/radiosensitivity via the activation of PI3K/Akt/mTOR signaling pathway (Ni et al., 2013). In breast and gallbladder carcinomas, the increased EpCAM expression is considered as a poor prognostic indicator (Shi et al., 2021; Prince et al., 2008). EpCAM is an important marker for cancer stem cells (CSCs) in the breast, prostate, pancreas, colon and hepatocellular cancers (Kaur et al., 2018; AbdelMageed et al., 2022; Dzobo et al., 2021; Zarębska et al., 2021). EpCAM can accelerate self-renewal and differentiation of CSCs and normal adult stem cells by directly targeting Wnt/β-catenin signaling pathway (Yamashita et al., 2007). The major malignant phenotypes of cancers (recurrence, metastasis, and chemoresistance) are attributable to the presence of CSCs, and thus, CSCs are now considered to be a pivotal target for diagnosis and therapy for many human cancers.

Development of monoclonal antibodies (mAbs) for cancer therapy has been growing rapidly over the past decades. Several anti-EpCAM mAbs were tested clinically for the treatment of different cancers. Edrecolomab (17-1A, Panorex) is an anti-EpCAM mAb and was approved for the adjuvant treatment of patients with resected colorectal cancer in Germany. However, phase III clinical data showed that it did not improve overall survival of patients with colon cancer and its marketing authorization was withdrawn (Fields et al., 2009). Catumaxomab (Removab) is a bispecific mAb against both EpCAM and CD3. It was approved by European Union in 2009 for the treatment of malignant ascites in patients with EpCAM^+^ carcinomas, but also withdrawn from the US market in 2013 and in the European Union market in 2017 due to commercial reasons (Jäger et al., 2012). Adecatumumab (MT201) is a mAb targeting EpCAM and was tested in patients with metastatic breast cancer in Europe. It showed dose- and target-dependent clinical activity in metastatic breast cancer, however, no objective tumor regression could be observed (Schmidt et al., 2010). ING-1 is another mAb targeting EpCAM and was tested in phase I in patients with advanced solid tumors including ovary, colon and lung cancers. The results showed that the risk of pancreatitis and the only marginal anti-tumor effect and may preclude further clinical monotherapy studies (Goel et al., 2007). Clinical studies so far with the anti-EpCAM mAbs for cancer therapy showed only the low success with limited anti-tumor effects, which highlights the need for the development of more efficacious anti-EpCAM antibody drug candidates.

Systemic application of mAbs in patients can be associated with the risk of high immunogenicity response, off-target and toxic side effects. Research has been actively looking for the other forms of antibodies replacing mAbs. A fully human single-domain antibody (sdAb) is an antibody fragment consisting of only a human variable heavy chain (VH) which can bind to an antigen (Deng et al., 2019; Velazquez et al., 2022; Gao et al., 2021). It has a small size of only 15 kDa compared to 150 kDa for a mAb, provides an efficient penetration into tumors and has no immunogenicity. Its small size enables its binding to hidden epitopes that are not accessible to conventional mAbs (Griffiths et al., 2016). It can also be easily manufactured in *E. coli* at a low cost. The human sdAbs against a variety of cancer cell surface proteins such as CXCR4, HER2 and mesothelin were successfully tested (Griffiths et al., 2016; Wang et al., 2019; Tang et al., 2013). Phage display technology can display a peptide on phage surface and is commonly used to screen antibodies from an antibody library (Jaroszewicz et al., 2022; Ghaderi et al., 2022). In this study, a critical EGF-like repeat epitope on the EpCAM extracellular domain surface was chosen for screening a human sdAb library by phage display. Five fully human anti-EpCAM sdAbs were identified and showed good *in vitro* and *in vivo* anti-tumor activities. They can potentially become new therapeutics for the treatment of various cancers.

## Materials and methods

### Cell culture

The human cell lines DU145, PC3 and MCF-7 were obtained from the American Type Culture Collection (ATCC, Rockville, MD, USA). DU145 and PC3 cells were cultured in RPMI 1640 medium (Gibco, Carlsbad, CA, USA), and MCF-7 in DMEM medium (Gibco). The media contained 10% fetal bovine serum (FBS, Gibco). Cells were cultured at 37 °C in 5% CO_2_ incubator.

### Selection of the anti-EpCAM phage sdAbs

A fully human sdAb phage library was obtained from Source BioScience (Nottingham, United Kingdom). The library contains the diversity in complementarity determining region 1 (CDR 1), CDR 2 and CDR3. SdAbs were displayed as fusion proteins on the surface of phage particles. SdAb phages were prepared from the library by the infecting *Escherichia coli* TG1 with KM13 helper phages according to the library instruction. The EpCAM fragment containing a critical EGF-like repeat epitope on the EpCAM protein surface was selected for panning the sdAb library. The amino acid sequence of this EGF-like repeat epitope is CAGRSSVSKVPVTVSCKCVDTQKT. Each Maxisorb tube (Nunc, New York, United States) was coated at 4°C overnight with 400 μg of the EPCAM fragment in 4 mL of phosphate-buffered saline (PBS). The tubes were washed three times with 4.5 mL of PBS and blocked with 4.5 mL of PBS containing 2% bovine serum albumin (BSA). After incubation at room temperature (RT) for 2 h, the tubes were washed three times with 4.5 mL of PBS, Then, 5 × 10^12^ sdAb library phages were added to each tube and incubated at RT for 1 h. The tubes were washed ten times with PBST (PBS containing 0.1% Tween 20). The remaining phages were eluted with 500 μL of Glycine-HCl (pH 2.2). Phages were propagated as described in the library instruction and used for the next round of panning. This process of panning was repeated for the four more rounds.

### Polyclonal phage ELISA

The polyclonal phages from each round of panning were analyzed by an enzyme-linked immunosorbent assay (ELISA). Each well of a 96-well plate (Nunc) was coated at 4°C overnight with the EpCAM fragment (0.2 μg/well). The wells were washed three times with PBS and blocked with 2% BSA. 10^10^ of amplified phage from each round of panning were added to each well. After incubation at RT for 1 h, the wells were washed three times with PBST. HRP (horseradish peroxidase)-conjugated anti-M13 antibody (1:10,000, Sino Biological, Beijing, China) was added to each well (100 μL/well) and incubated at RT for 1 h. The wells were washed three times with PBST. TMB substrate (3, 3′, 5, 5′-Tetramethylbenzidin) (Beyotime Biotech., Shanghai, China) was added to each well (100 μL/well). After incubation for 5 min, the reaction was stopped with 50 μL of 1M H_2_SO_4_. Absorbance was read at 450 nm by a plate reader (Bio-RAD 680, Bio-RAD, Hercules, CA, United states).

### Monoclonal phage ELISA

The phages from the fifth round of panning showing the highest absorbance among the five rounds of panning in polyclonal phage ELISA were used for selection of monoclonal anti-EpCAM sdAb phages. *E. coli* TG1 was infected with the eluted phages from the fifth round of panning and plated on TYE agar containing 100 μg/mL ampicillin and 1% glucose. Then, 478 colonies were randomly picked and cultured in 96-well plates at 37°C and 220 rpm overnight. Then, 5 μL of the bacterial culture were added to 200 μL of 2 × TY medium and incubated at 37°C and 250 rpm. When OD600 of 0.5 was reached, KM13 helper phages were added and the culture was incubated at 37°C for 0.5 h. The culture was centrifuged at 3,200 g, and the pellet was resuspended in 2 × TY containing 100 μg/mL Ampicillin and 50 μg/mL Kanamycin. The culture was propagated at 25°C and 250 rpm for 20 h and centrifuged at 3,200 g for 10 min. The supernatant containing the sdAb phages was used for monoclonal phage ELISA. Monoclonal phage ELISA was performed as described above for polyclonal phage ELISA. The phage clones of good antibody specificity and high absorbance were sequenced. The nucleotide sequences of the phage clones were compared by the DNAMAN software to identify the different anti-EpCAM sdAbs.

### Expression and purification of the soluble anti-EpCAM sdAb proteins

The different anti-EpCAM sdAb phage clones were amplified by polymerase chain reaction (PCR), and the PCR product was cloned into pET-22b vector (Novagen, Madison, WI, United States) by Not I and Nco I restriction endonuclease sites. The plasmid was transformed into *E. coli* BL21 (Novagen). A bacterial colony for each sdAb clone was incubated in LB medium at 37°C and 230 rpm. When OD600 of 0.8 was reached, isopropyl β-D-thiogalactopyranoside (IPTG, Sangon Biotech, Shanghai, China) was added at a final concentration of 0.5 mM, and the culture was incubated at 25°C and 230 rpm for 6 h. It was centrifuged, and the pellet was resuspended in PBS containing phenylmethylsulfonyl fluoride (PMSF, Sangon Biotech). Bacteria were broken down by the sonication, and the bacterial suspension was centrifuged at 4°C and 15000g for 30 min. The supernatant containing the soluble sdAb proteins was purified using nickel nitrilotriacetic acid (Ni-NTA) column (Sevensea Biotech, Shanghai, China). The purity of the extracted soluble sdAbs was determined by 12% SDS-PAGE (sodium dodecyl sulfate polyacrylamide gel electrophoresis).

### ELISA analysis with the purified anti-EpCAM sdAb proteins

To detect specificity of the anti-EpCAM sdAbs to bind to EpCAM, 0.2 μg of EpCAM fragment or EpCAM complete extracellular domain protein (Shanghai Bootech BioSci. and Technol., Shanghai, China) was coated in the wells of a 96-well plate (Nunc) at 4°C overnight. After washing and blocking the wells, 100 μL of purified anti-EpCAM sdAb at a concentration of 1 μg/mL was added to each well. Notably, the sdAbs utilized in this study were non-Fc-fused. Protein A was employed to bind to the variable regions of the sdAbs, facilitating detection through HRP-conjugated Protein A. After the wells were incubated at RT for 1 h, 100 μL of HRP-conjugated protein A (1:5,000, Thermo Fisher, Waltham, MA, USA) was added. After the wells were incubated at RT for 1 h and washed with PBST, 100 μL of TMB substrate (Beyotime Biotech.) was added to each well. After incubation for 5 min, the reaction was stopped with 50 μL of 1 M H_2_SO_4_. Absorbance was read at 450 nm.

### Cell binding analysis by flow cytometry

Cells (1 × 10^6^/mL) were incubated with the different anti-EpCAM sdAbs (experimental groups), or the negative control sdAbs at 4°C for 1 h. A mouse anti-EpCAM mAb (positive control group) or an isotype control mAb (Santa Cruz Biotechnology, Dallas, TX, USA) was included. Cells were washed with PBS. For the experimental groups and the negative control sdAbs, cells were stained using fluorescein isothiocyanate (FITC)-conjugated protein A (Abcam, Cambridge, MA, United States). For positive control group or an isotype control mAb, cells were stained using phycoerythrin (PE)-conjugated mouse IgG kappa binding protein (Santa Cruz Biotechnology). Notably, the sdAbs used in this analysis are non-Fc-fused. Protein A was utilized to bind specifically to the variable regions of the sdAbs, enabling detection through FITC-conjugated Protein A. Cell staining was analyzed by a FACS Calibur (BD Biosciences, San Jose, CA, United States). FlowJo software (BD Biosciences) was used for data analysis.

### Cell viability assay

Cells were incubated in 96-well plates (5,000 cells/well) at 37°C overnight in culture medium containing 10% FBS. The next day, the medium was replaced with serum-free medium, and the cells were starved for 4 h. Following starvation, the cells were treated with various concentrations of sdAbs (25 μg/mL, 50 μg/mL and 100 μg/mL) for 72 h. They were incubated at 37°C for 4 h with 20 μL/well of 5 mg/mL 3-(4,5-Dimethylthiazol-2-yl)-2,5 diphenyltetrazolium bromide (MTT, Sigma, St. Louis, MO, United States). The medium was replaced by 150 μL of dimethyl sulfoxide (DMSO, Sigma), and the optical density (OD) was determined at 570 nm on a plate reader (Bio-RAD).

Cells were seeded in 96-well plates at a density of 5,000 cells per well and incubated overnight at 37°C in culture medium containing 10% fetal bovine serum (FBS). The next day, the medium was replaced with serum-free medium, and the cells were starved for 4 h. Following starvation, the cells were treated with various concentrations of sdAbs (e.g., 1 μg/mL, 5 μg/mL, 10 μg/mL) in serum-free medium and incubated for 72 h at 37°C.

After 72 h of incubation, 20 μL of 5 mg/mL 3-(4,5-Dimethylthiazol-2-yl)-2,5-diphenyltetrazolium bromide (MTT, Sigma, St. Louis, MO, USA) was added to each well, and the plates were incubated for an additional 4 h at 37°C. The medium was then carefully removed, and 150 μL of dimethyl sulfoxide (DMSO, Sigma) was added to each well to dissolve the formazan crystals. The optical density (OD) was measured at 570 nm using a plate reader (Bio-RAD).

### Cell apoptosis analysis

Cells were incubated at 37°C overnight in culture medium containing 10% FBS in 6-well plates (5 × 10^5^ cells/well) and starved in a serum-free medium for 4 h. Cells were cultured for 48 h with fresh medium containing 50 μg/mL sdAbs or PBS as a control. They were trypsinized, washed with PBS and suspended at a concentration of 2 × 10^6^ cells/mL. Cells were stained in the dark for 15 min with FITC-conjugated Annexin V (5 μL) and propidium iodide (PI, 10 μL) (Sangon Biotech). Cell fluorescence was detected by a FACS Calibur (BD Biosciences). FlowJo software (BD Biosciences) was used for data analysis.

### Scratch assay

Cells were incubated overnight in culture medium containing 10% FBS in a 12-well plate (2 × 10^5^ cells/well). When cells reached 90% confluence, they were starved in a serum-free medium for 4 h. Cells were scratched using a 200 μL pipette tip and washed to remove cell debris. They were incubated in culture medium containing 1% FBS and the sdAbs for 24 h. Cell photographs were taken using a microscope (Nikon, Tokyo, Japan). Wound widths were determined by Image-Pro Plus 6.0 software (Media Cybernetics, Rockville, MD, United States). Cell migration rate was determined by Lm = (L0 − Lt)/L0 × 100% in which Lm is cell migration rate, L0 is wound width at 0 h, and Lt is wound width at 24 h.

### Cell migration and invasion assays

For the cell migration assay, 2 × 10^4^ cells were resuspended in 200 μL of culture medium containing 1% FBS and the sdAbs and seeded in the upper transwell chamber (8 μm pore size, BD Biosciences) placed in a 24-well plate. Then, medium containing 20% FBS was added to each well, and cells were incubated for 24 h. Non‐migrated cells in the upper chamber were removed using a cotton swab, and migrated cells on the bottom surface of the membrane were fixed with 4% paraformaldehyde (Sigma) and stained with 0.1% crystal violet (Beyotime Biotech). Cell photographs were taken using a microscope (Nikon). Cells were stained with crystal violet, which was dissolved in 33% acetic acid solution, and absorbance was read at 570 nm. Cell invasion assay was performed similarly, except that 30 μL of matrigel (BD Biosciences) was added to upper chamber before cells were seeded.

### Xenograft tumor model

Animal study procedures were approved by the Institutional Animal Care and Use Committee of Jinan University. DU145 cells (5 × 10^6^ cells) were subcutaneously inoculated into right flank of each 4-week-old male BALB/c nude mouse (Guangdong Medical Experimental Animal Center, Guangzhou, China). Mice were intravenously administered every 3 days with the sdAbs (10 mg/kg) or cis-platinum (DDP, 2 mg/kg) or PBS as a control when tumors reached about 100 mm^3^. DDP was purchased from the pharmacy of the first affiliated hospital of Jinan University (Guangzhou, China). Tumor volumes were determined (0.5 × length × width^2^). Mice were sacrificed at the end of the experiment, and tumors were isolated.

### Immunohistochemical staining

Tumors were fixed using formaldehyde and embedded in paraffin and cut into 4 μm sections. Sections were stained with hematoxylin-eosin (HE, Beyotime Biotech) or incubated at 4°C overnight with anti-Ki67, anti-CD31 and anti-cleaved caspase-3 (c-caspase-3) (1:200, Sigma). After washing three times with PBS, they were incubated at 37°C for 1 h with HRP-conjugated goat anti-rat antibody (Sigma). After washing three times with PBST, they were incubated with diaminobenzidine (DAB) chromogen (Sigma) for 4 min. Photographs were taken using an Olympus IX70 microscope (Olympus, Tokyo, Japan). The integrated optical density (IOD) of each graph was determined for a quantitative measure of staining intensity using Image-Pro Plus software (Media Cybernetics).

### Statistical analysis

Data shown in the study were obtained in at least three independent experiments performed in a parallel manner unless otherwise indicated. All values were presented as mean ± standard deviation (SD). Graphs were plotted using GraphPad Prism 8 software (Graphpad, La Jolla, CA, United States). The differences were determined using one-way ANOVA. *P* < 0.05 was considered statistically significant.

## Results

### Screening for the anti-EpCAM sdAbs in a fully human sdAb phage library

**FIGURE 1:**
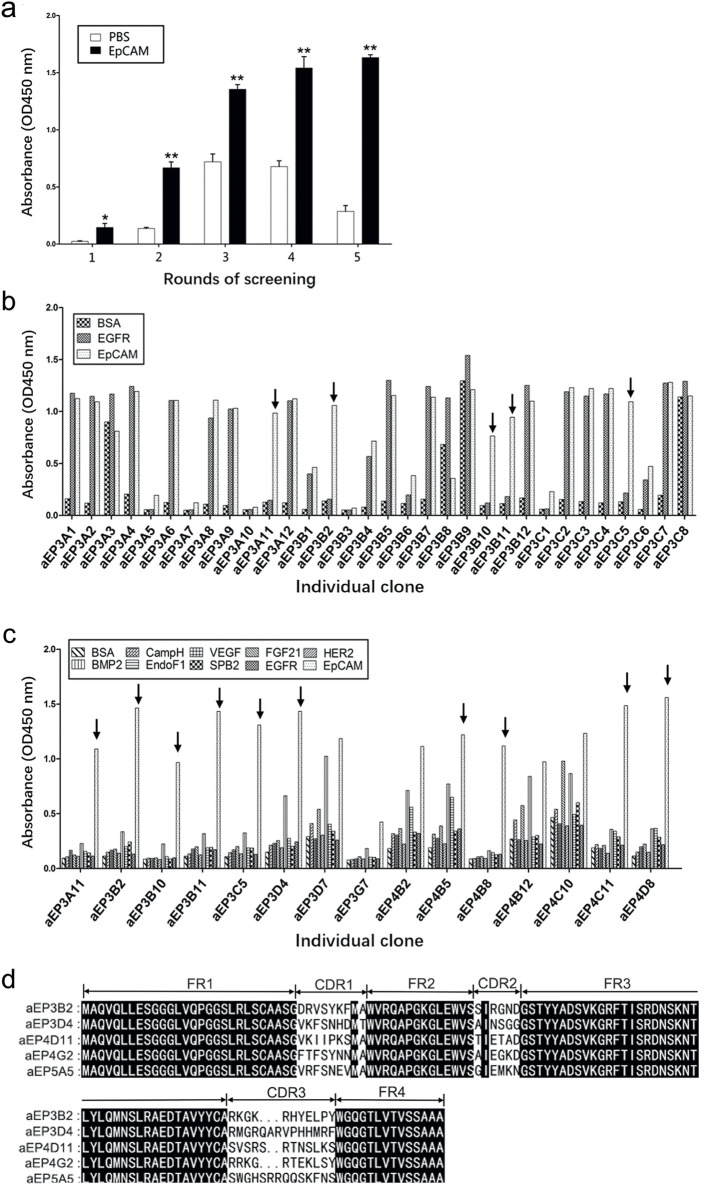
Five anti-EpCAM sdAbs are screened out from a phage display sdAb library. **(A)** Phages from each round of panning were monitored against EpCAM peptide by polyclonal phage ELISA. PBS was used as a negative control. The values are the mean ± SD (n = 5). *P < 0.05 and **P < 0.01 versus the respective PBS control. **(B)** Phage clones were screened using monoclonal phage ELISA. A total of 478 phage clones from the fifth round of panning were tested for binding to EpCAM peptide, and data for the representative 32 clones are shown. The Arrows indicate the clones which bound to EpCAM only and not to BSA and EGFR as negative controls. **(C)** The phage clones were tested using monoclonal phage ELISA, and the results of the representative 15 clones were shown. The Arrows indicate the clones which specifically bound to EpCAM only and not to the other 9 proteins as negative controls. **(D)** Five anti-EpCAM phage clones were identified. Amino acid sequence alignment of 5 clones is shown. Amino acid sequences were derived from the respective nucleotide sequences by DNAMAN software. CDR, complementarity determining region; FR, framework region.

To enrich for the anti-EpCAM sdAbs, five successive rounds of screening of a fully human sdAb phage library were performed with an EpCAM peptide. The result of each round of screening was shown (Table 1), and the enrichment ratio (P/N) increased to 59.36 after the fifth round of screening. Phages derived from each round of screening were tested for binding to the EpCAM peptide by polyclonal phage ELISA. The results showed that binding to the EpCAM peptide increased along with each round of screening (Figure 1A).

**TABLE 1:** Enrichment of anti-EpCAM sdAb phages from screening a phage library.

Round	Antigen (μg/mL)	Input phage (pfu)	Output phage (pfu) (p)	Output phage of negative control (pfu) (N)	Recovery Rate (P/input phage)	P/N
1	100	5 × 10^12^	4.51 × 10^5^	1.19 × 10^5^	9.02 × 10^−8^	3.79
2	50	5 × 10^12^	2.70 × 10^7^	2.46 × 10^6^	5.40 × 10^−6^	10.98
3	50	5 × 10^12^	2.98 × 10^8^	8.24 × 10^6^	5.96 × 10^−5^	36.17
4	25	5 × 10^12^	1.19 × 10^9^	2.52 × 10^7^	2.38 × 10^−4^	47.22
5	25	5 × 10^12^	5.39 × 10^9^	9.08 × 10^7^	1.07 × 10^−3^	59.36

*Pfu*, plaque forming unit; *P*, positive; *N*, negative.

A total of 478 clones were randomly picked from the fifth round of screening and analyzed for binding to the EpCAM peptide by monoclonal phage ELISA. The results of the representative 32 clones were shown (Figure 1B). Thirty phage clones could bind to the EpCAM peptide and not to BSA and EGFR as the negative controls. These 30 clones were further analyzed by monoclonal phage ELISA for binding to the EpCAM peptide and the nine unrelated proteins as negative controls. The 17 phage clones could bind to the EpCAM peptide but not to the other nine unrelated proteins. Results of the representative 15 clones were shown (Figure 1C). DNA sequencing and analysis revealed that some phage clones were the same, and the five different human anti-EpCAM sdAbs were identified and named aEP3B2 (accession number: LR535669), aEP3D4 (accession number: LR535670), aEP4D11 (accession number: LR535671), aEP4G2 (accession number: LR535673) and aEP5A5 (accession number: LR535672), respectively. The comparison of their amino acid sequences derived from their DNA sequences was showed (Figure 1D). These five human sdAbs share the four same framework regions (FR1-4) and have three different complementarity determining regions (CDR1-3).

### Expression and analysis of the five anti-EpCAM sdAbs

**FIGURE 2:**
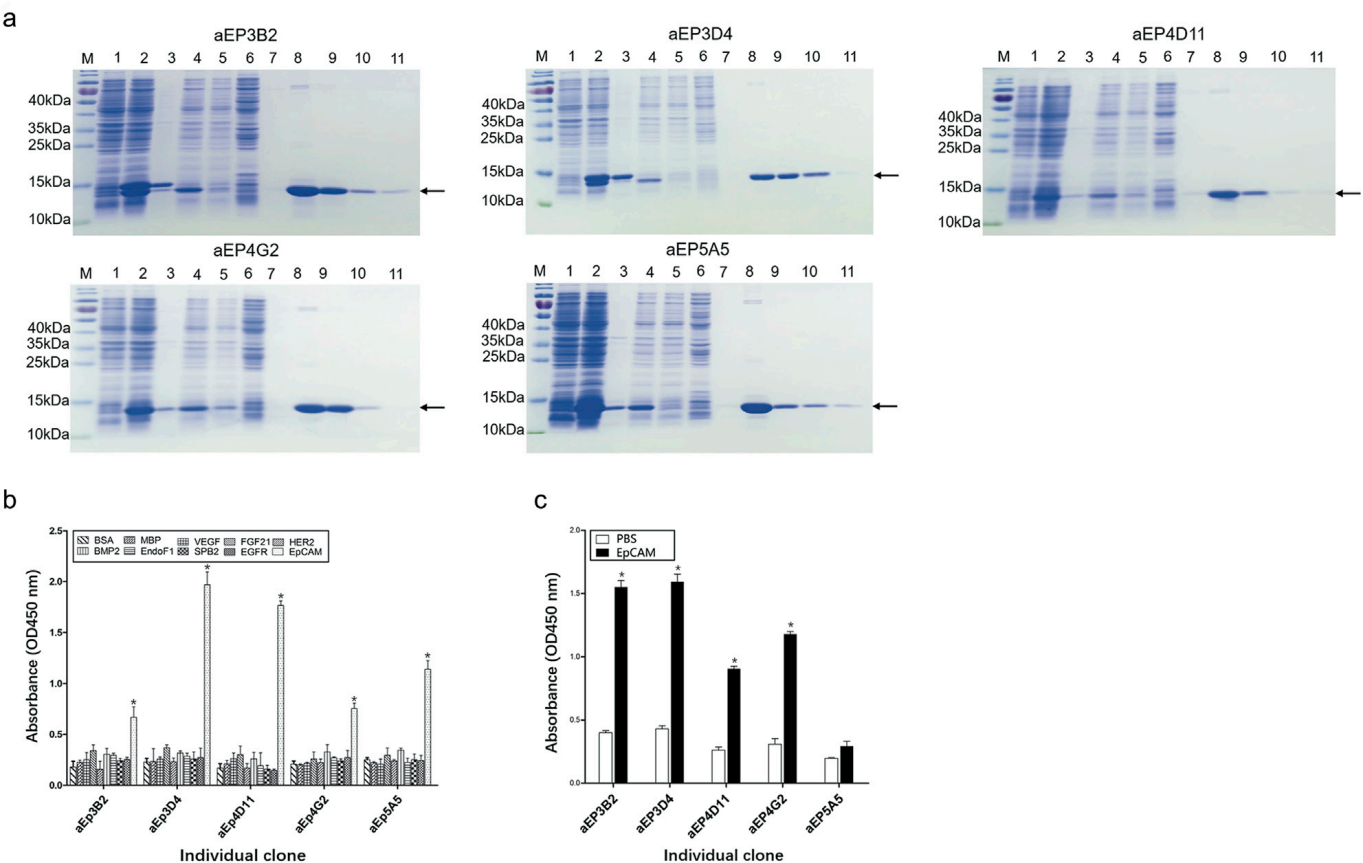
Purification and analysis of the five anti-EpCAM sdAbs. **(A)** Expression and purification of the anti-EpCAM sdAbs were analyzed by SDS-PAGE. M protein markers; lane 1: total bacterial lysate before IPTG induction; lane 2: total bacterial lysate after IPTG induction; lane 3: pellet of induced bacterial lysate after sonication; lane 4: supernatant of induced bacterial lysate after sonication; lane 5: flow-through portion from the Ni-NTA column after soluble bacterial lysate was added; lane 6: flow-through portion from the column after washing buffer was added; lanes 7–11: fractions from the column after elution buffer was added. Arrows indicate the anti-EpCAM sdAbs. **(B)** Binding of the purified sdAbs to the EpCAM peptide was determined by ELISA. The purified sdAbs specifically bound to the EpCAM peptide, and not to the nine other irrelevant proteins as negative controls. **(C)** Binding of the purified sdAbs to the EpCAM complete extracellular domain was determined by ELISA. PBS was included as a control. The values are the mean ± SD (n = 5). *P < 0.05 versus the respective BSA (**b**) or PBS control (**c**).

The five human anti-EpCAM sdAbs were expressed in *E. coli* BL21 (DE3), and the sdAbs in soluble fraction after bacterial breakage were purified using Ni-NTA resin column. Each purified sdAb showed a single band marked by an arrow (Figure 2A).

**FIGURE 3:**
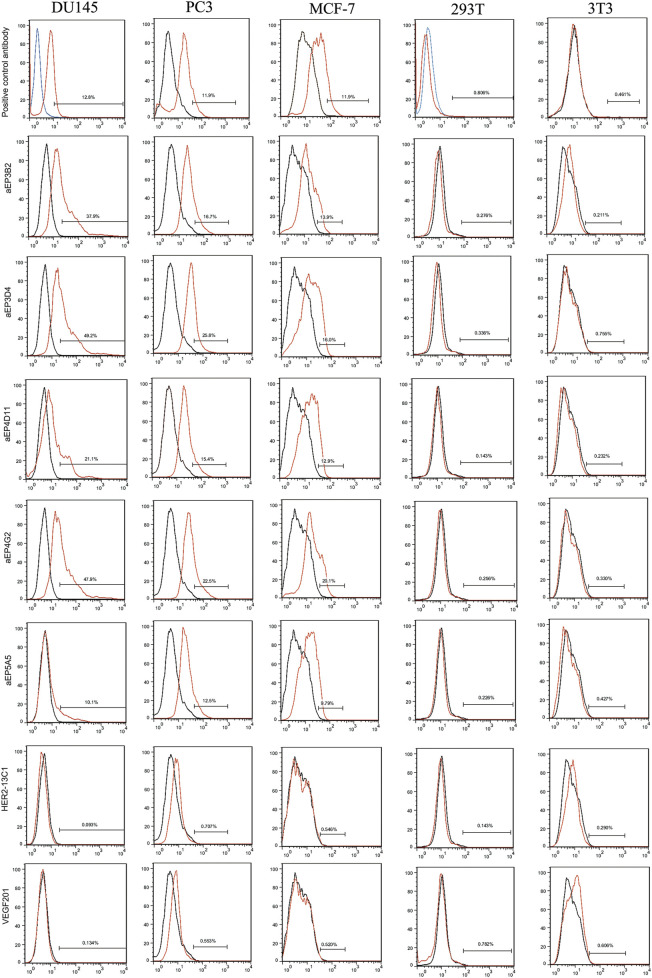
Flow cytometry analysis shows the specific binding of the anti-EpCAM sdAbs to cancer cells. The 293T and 3T3 cell lines were tested as controls. For the five anti-EpCAM sdAbs and the two negative control sdAbs (HER2-13C1 and VEGF201), binding was visualized with FITC-conjugated protein A. For a mouse anti-EpCAM mAb (a positive control) or an isotype control mAb, binding was visualized with PE-conjugated mouse IgG kappa binding protein. Red curves represent the cells incubated with the five anti-EpCAM sdAbs or the two negative control sdAbs or the mouse anti-EpCAM mAb. Black and blue curves represent the cells incubated with an isotype control mAb.

To confirm if the five purified anti-EpCAM sdAbs could specifically bind to EpCAM, ELISA was performed to examine their binding to the EpCAM peptide and the nine unrelated proteins as negative controls. Results showed that the five sdAbs could bind to the EpCAM peptide, but, not to the nine unrelated antigens, and two (aEP3D4 and aEP4D11) of them gave higher absorbance than the others (Figure 2B). These results indicated that the five purified sdAbs retained the binding specificity of their respective phage clones. ELISA was also performed to examine the binding of these five sdAbs to the EpCAM complete extracellular domain purchased commercially. Results showed that four sdAbs (aEP3B2, aEP3D4, aEP4D11 and aEP4G2) could also bind to the EpCAM complete extracellular domain, and two sdAbs (aEP3B2 and aEP3D4) gave the higher absorbance than the others (Figure 2C). Furthermore, binding of the five anti-EpCAM sdAbs to the three cancer cell lines DU145, PC3 and MCF-7 was tested by flow cytometric analysis, and 293T and 3T3 cells were included as negative controls. The results showed that the five sdAbs could bind to the three cancer cell lines, but not to 293T and 3T3 cells (Figure 3). These data indicate that the sdAbs can specific target cancer cells via direct binding to the EpCAM.

### The anti-EpCAM sdAbs inhibit cancer cell proliferation and induce cancer cell apoptosis

**FIGURE 4:**
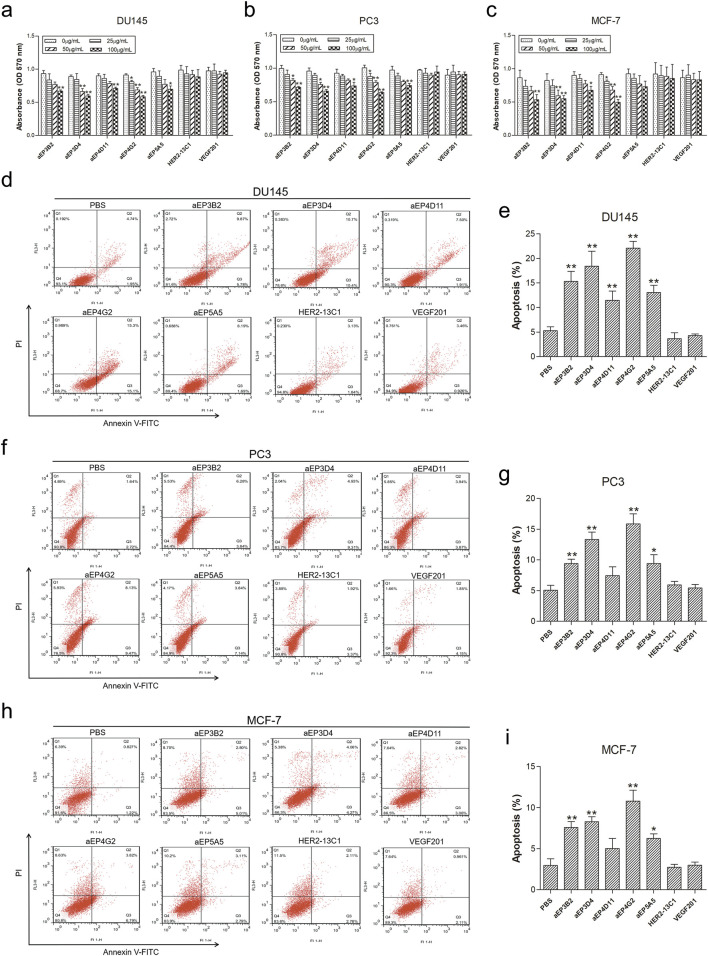
The anti-EpCAM sdAbs decrease viability and induce apoptosis in cancer cells. **(A–C)** For cell viability assay, DU145 **(A)**, PC3 **(B)** and MCF-7 **(C)** cells were incubated for 72 h with increasing concentrations of the five anti-EpCAM sdAbs or the two negative control sdAbs (HER2-13C1 and VEGF201). **(D–I)** For cell apoptosis analysis, DU145 **(D, E)**, PC3 **(F, G)** and MCF-7 **(H, I)** cells were treated for 48 h with 50 μg/mL anti-EpCAM sdAbs or the two negative control sdAbs or PBS. Annexin V-FITC can detect cell apoptosis (%) at an early stage. Propidium iodide (PI) can distinguish viable from non-viable cells. The values are the mean ± SD (n = 5). *P < 0.05 and **P < 0.01 versus the respective control (0 μg/mL) **(A–C)** or PBS control **(D–I)**.

MTT assays were performed to evaluate effects of the five anti-EpCAM sdAbs on the proliferation of DU145, PC3 and MCF-7 cells. Cells were cultured for 72 h with different concentrations of the purified sdAbs. All five sdAbs showed the inhibition on the growth of the three cancer cell lines (Figures 4A–C). More inhibition was seen on three cell lines at the highest sdAb concentration (100 μg/mL). Two sdAbs (HER2-13C1 and VEGF201) were isolated previously in our laboratory in a different study from the same fully human sdAb phage library (previously unpublished data) and could not bind to EpCAM. Their proteins were purified with the same method as the five anti-EpCAM sdAbs. They were included as negative controls and did not show the inhibition of these three cell lines.

Apoptosis assay was performed with Annexin V-FITC and PI to evaluate effects of the five anti-EpCAM sdAbs on cell apoptosis. For DU145 cells, all five sdAbs could significantly increase cell apoptosis compared with PBS and the negative control sdAbs (HER2-13C1 and VEGF201) (Figures 4D, E). For PC3 and MCF-7 cells, only four sdAbs (aEP3B2, aEP3D4, aEP4G2 and aEP5A5) significant increased cell apoptosis (Figures 4F–I).

### The anti-EpCAM sdAbs inhibit cancer cell migration and invasion

**FIGURE 5:**
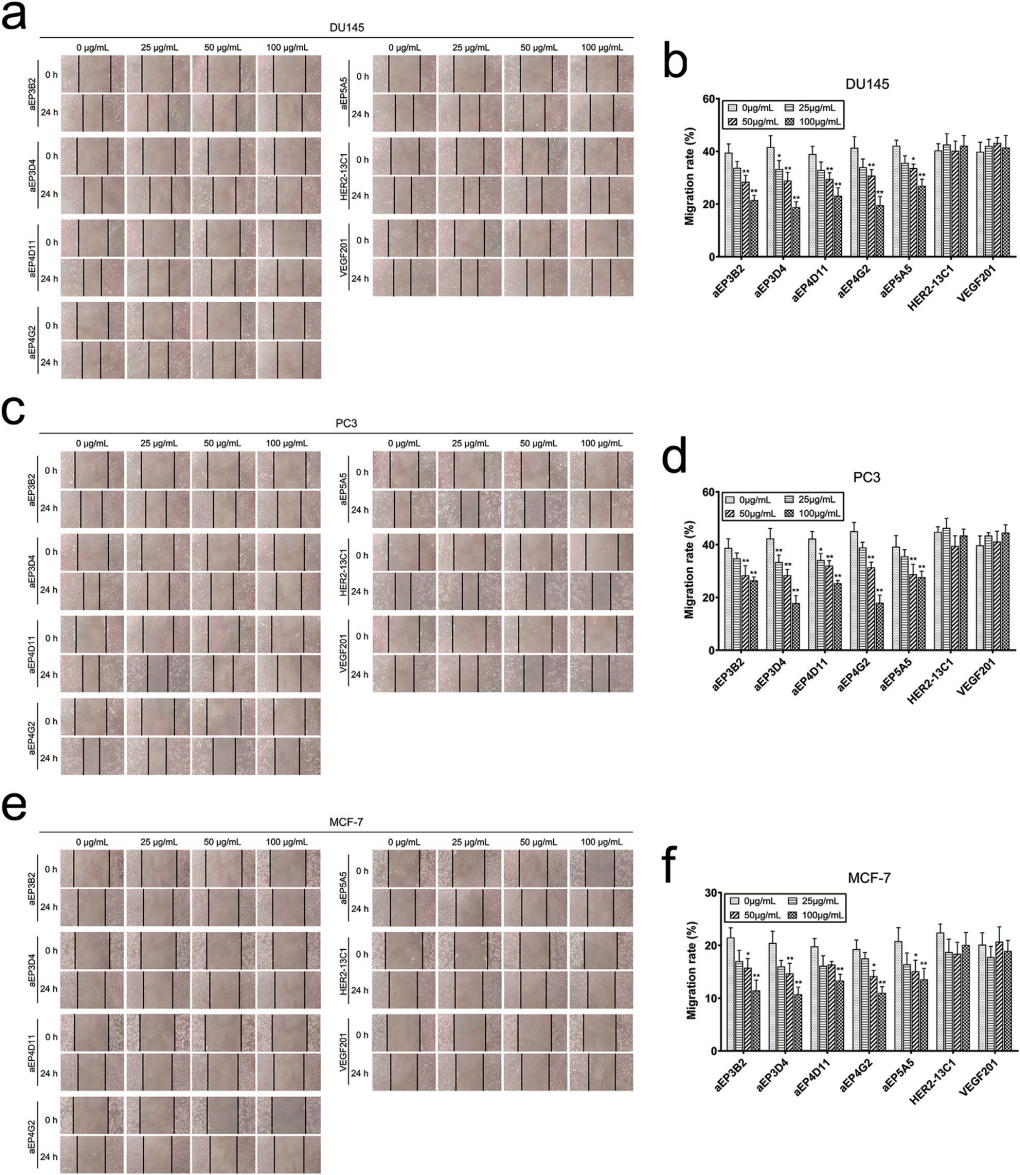
The anti-EpCAM sdAbs reduce cancer cell migration in scratch assay. After cell monolayers were scratched, DU145 **(A, B)**, PC3 **(C, D)** and MCF-7 **(E, F)** cells were incubated for 24 h with increasing concentrations of the five anti-EpCAM sdAbs or the two negative control sdAbs. Images were captured at 0 h and 24 h post scratching. The scratch gaps were indicated by the vertical lines. Cell migration rates (%) were calculated for each cancer cell line. The values are the mean ± SD (n = 5). *P < 0.05 and **P < 0.01 versus the respective control (0 μg/mL).

**FIGURE 6:**
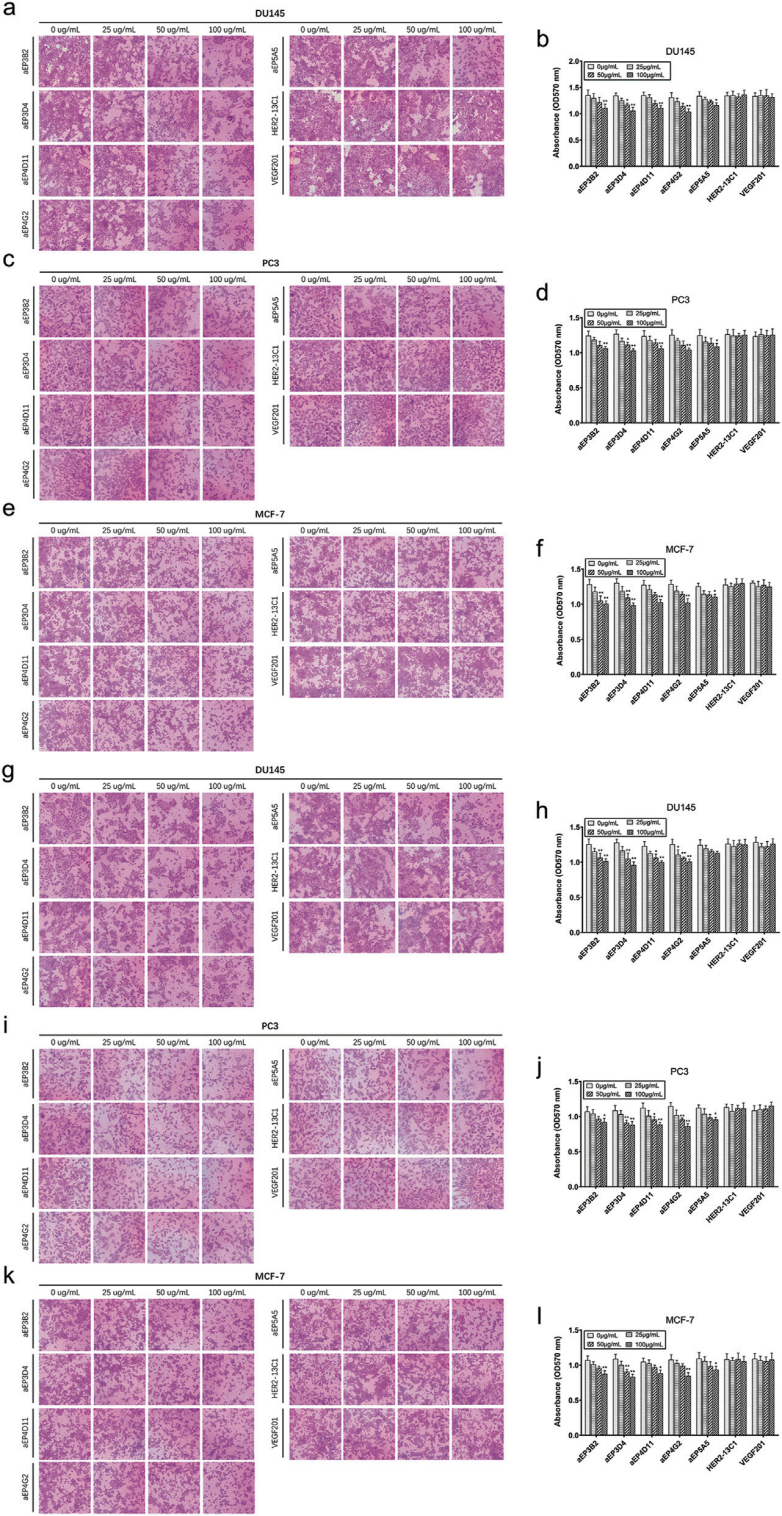
The anti-EpCAM sdAbs inhibit cancer cell migration and invasion. a-**f** For cell migration assay, DU145 **(A, B)**, PC3 **(C, D)** and MCF-7 **(E, F)** cells were resuspended in culture medium containing 1% FBS and the five anti-EpCAM sdAbs or the two negative control sdAbs and seeded in the upper transwell chamber placed in a 24-well plate. The medium containing 20% FBS was added to each well, and cells were incubated for 24 h. Photographs of the migrated cells on the bottom surface of the membrane were taken. Cells were stained with crystal violet and dissolved in 33% acetic acid solution, and absorbance was read at 570 nm. **(G–L)** For cell invasion assays, DU145 **(G, H)**, PC3 **(I, J)** and MCF-7 **(K, L)** cells were treated similarly, except that 30 μL of matrigel was added to upper chamber before cells were seeded. The values are the mean ± SD (n = 5). *P < 0.05 and **P < 0.01 versus the respective control (0 μg/mL).

Cell scratch assay was performed to examine effect of the anti-EpCAM sdAbs on cancer cell migration. Cells were cultured with different concentrations of the purified sdAbs for 24 h after making the scratches on cell monolayers. All five sdAbs showed the inhibition of cell migration of the three cancer cell lines (DU145, PC3 and MCF-7) (Figures 5A–F). The inhibition of cell migration was generally sdAb concentration-dependent, and the higher sdAb concentrations caused more inhibition of cell migration. Two sdAbs (HER2-13C1 and VEGF201) as negative controls did not show the inhibition of cell migration of these three cell lines. Transwell assay was also performed to examine effect of the five anti-EpCAM sdAbs on cancer cell migration. Results also showed that the five sdAbs inhibited cell migration of the three cancer cell lines (Figures 6A–F). Less inhibition of cell migration was seen by aEP5A5 for all three cell lines. Transwell assay was also performed to examine effect of the five anti-EpCAM sdAbs on cancer cell invasion using the transwells coated with matrigel. Data showed that the five sdAbs could decrease cell invasion of the three cell lines (Figures 6G–L). Less inhibition of cell invasion was seen by aEP5A5 for all three cell lines.

### The anti-EpCAM sdAbs inhibit tumor grouth *in vivo*

**FIGURE 7:**
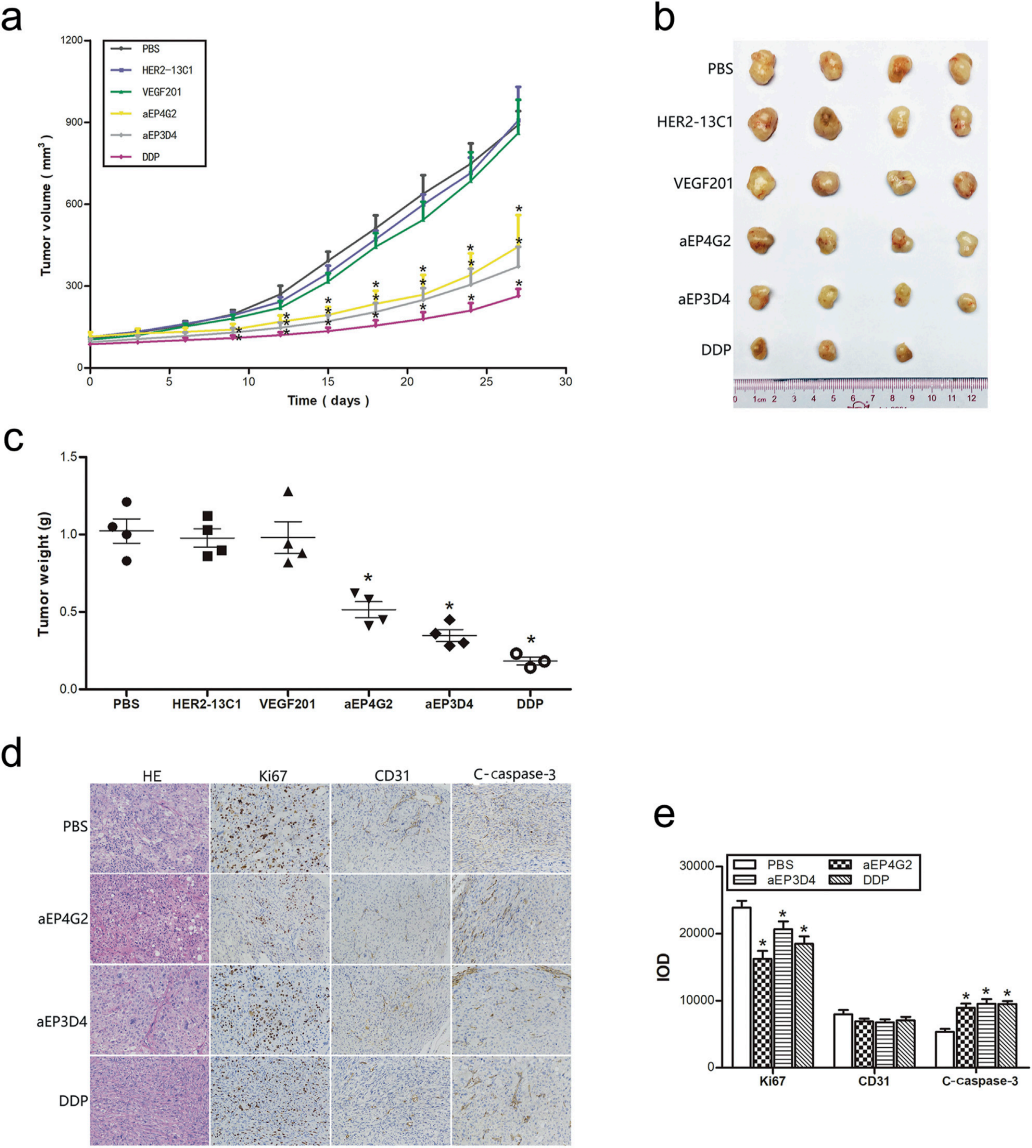
The anti-EpCAM sdAbs inhibit tumor growth *in vivo*. **(A–C)** The mice bearing xenograft tumors were administrated intravenously every 3 days with the anti-EpCAM sdAbs (aEP3D4 and aEP4G2) or the negative control sdAbs (HER2-13C1 and VEGF201). PBS and DDP were administrated as controls. Tumor volumes were monitored every 3 days. The tumors were excised, weighed and photographed on the 27th day. **(D–E)** The anti-EpCAM sdAbs inhibit Ki67 expression for cell proliferation, increase c-caspase-3 expression for cell apoptosis *in vivo* and have no effect on the CD31 expression for tumor angiogenesis. Tumor sections were stained by HE. For immunohistochemistry, they were checked with antibodies against Ki67, CD31 and c-caspase-3. The integrated optical density (IOD) of each graph was determined for a quantitative measure of staining intensity using Image-Pro Plus software. The values are the mean ± SD. *P < 0.05 and **P < 0.01 versus the respective PBS control.

The *in vitro* cell functional studies described above consistently showed that more inhibition of cell functions was seen with aEP3D4, aEP4G2 and DU145 cells. Therefore, a mouse tumor xenograft model was established with DU145 cells to study effect of aEP3D4 and aEP4G2 on xenograft growth. When the tumors reached an average volume of 100 mm^3^, mice were injected intravenously with PBS, DDP (2 mg/kg) as a control, anti-EpCAM sdAbs (aEP3D4 and aEP4G2, 10 mg/kg) and the negative control sdAbs (HER2-13C1 and VEGF201, 10 mg/kg) every 3 days. The tumor volumes were significantly reduced on day 27 following the first injections of the test reagents in aEP3D4, aEP4G2 and DDP groups compared with PBS or the two negative control sdAb groups (Figures 7A, B). The two negative control sdAbs gave tumor volumes similar to PBS. Tumor weights were also significantly reduced in aEP3D4, aEP4G2 and DDP groups compared with PBS or the two negative control sdAb groups (Figure 7C).

To preliminarily study the mechanisms how the anti-EpCAM sdAbs inhibit cancer cell growth *in vivo*, immunohistochemistry was performed with the xenografts removed from the mouse xenograft model. Anti-Ki67 antibody was used for detecting the tumor cell proliferation, anti-CD31 antibody for tumor angiogenesis and anti-c-caspase-3 antibody for tumor cell apoptosis (Figures 7D, E). Cell proliferation was significantly lower in aEP3D4, aEP4G2 and DDP groups than PBS. Cell apoptosis was significantly higher in aEP3D4, aEP4G2 and DDP groups than PBS. No significant difference was detected for tumor angiogenesis in all groups.

## Discussion

Traditional cancer therapies, such as surgical resection, chemotherapy and radiotherapy, commonly result in low cure rates. Antibody-based medicines have the potential to provide high specificity and more effective treatment. In the past years, some new anti-cancer antibodies have been successfully developed to enable tumor regression or malignancy attenuation (Arlotta and Owen, 2019; Shin et al., 2021). EpCAM is a homophilic cell-cell adhesion glycoprotein and is expressed on CSCs in epithelial tumors and circulating tumor cells (Kalantari et al., 2022; Hwang et al., 2022). Several anti-EpCAM mAbs have been clinically tested for cancer therapy. However, the low efficacy of anti-EpCAM mAbs in clinical trials needs to develop the better kinds of antibodies. The fully human sdAbs can be good alternatives.

An epitope located on the EpCAM extracellular domain was chosen as the antigen for screening of the human anti-EpCAM sdAbs by phage display. According to the crystal structure of the EpCAM extracellular domain, the epitope is located on the surface of the EpCAM extracellular domain, which is accessible for binding to antibody (Pavšič et al., 2014). The epitope is relatively independent of the other EpCAM amino acids in the three-dimensional structure. The secondary structure of the synthesized EpCAM epitope is very similar to that of the intact EpCAM extracellular domain. Therefore, this EpCAM epitope can be good for screening of human sdAbs that may bind to the natural EpCAM protein. In addition, this EpCAM epitope is an epidermal growth factor (EGF)-like repeat, which is quite similar to the EGF-like motif in the rod domain of nidogen (Schnell et al., 2013). Nidogen is a basement membrane glycoprotein involved in cell-matrix adhesion, which implies that this EpCAM epitope may be necessary to maintain the EpCAM homophilic cell-cell adhesion function. So, this EpCAM epitope may contribute to the potent anti-tumor effect of our new anti-EpCAM sdAbs.

In this study, an EpCAM peptide was used to screen the anti-EpCAM sdAbs from a fully human sdAb phage library. Five human anti-EpCAM sdAbs were isolated. ELISA showed that all five sdAbs could specifically bind to the EpCAM peptide (Figure 2B), and four of them could also bind to the EpCAM complete extracellular domain purchased commercially (Figure 2C). Flow cytometric analysis showed that all five sdAbs could also specifically bind to the three cancer cell lines, but not to 293T and 3T3 cells as negative controls (Figure 3). In consistency with ELISA results (Figure 2C), aEP5A5 also showed the least binding to the three cancer cell lines (Figure 3). EpCAM was over-expressed in primary prostate tumors and lymph node metastases and was associated with prostate cancer cell proliferation, invasion and metastasis (Ni et al., 2013). Furthermore, the enhanced EpCAM expression could be considered as a poor prognostic marker in breast carcinomas (Kaur et al., 2018). So, anti-tumor effect of the five human anti-EpCAM sdAbs was evaluated with three EpCAM^+^ cancer cell lines (DU145, PC3 and MCF-7). MTT and cell scratch assays showed that the five sdAbs could inhibit the proliferation and migration of the three cancer cell lines (Figures 4A–C, 5). The five sdAbs could increase the apoptosis of the three cancer cell lines (Figures 4D–I). In addition, transwell assay also showed that the five sdAbs could inhibit the migration and invasion of the three cancer cell lines (Figure 6). Two anti-EpCAM sdAbs (aEP3D4 and aEP4G2) were evaluated for their anti-tumor effect *in vivo* and could inhibit tumor growth in a mouse tumor xenograft model. These results clearly demonstrate that these human sdAbs have good anti-tumor effects both *in vitro* and *in vivo* and are good candidates for cancer therapy. Notably, in our *in vivo* experiments, the sdAbs achieved a tumor volume reduction of approximately 70% compared to the PBS control, a result comparable to that observed with the positive control, cisplatin. However, benchmarking the performance of our sdAbs against best-in-class anti-EpCAM antibodies, such as adecatumumab or edrecolomab, would provide further insights into their therapeutic potential. While sdAbs offer theoretical advantages, including smaller size for enhanced tumor penetration and fully human sequences to minimize immunogenicity, direct comparisons with established antibodies would enhance the robustness of our findings.

Previous studies showed that EpCAM intracellular domain could promote tumorigenesis in tumor initiation cells (TICs) through the up-regulation of reprogramming genes and the epithelial-mesenchymal transition (EMT), and release of its extracellular domain could further enhance EpCAM cleavage and trigger its intracellular domain-mediated signaling in an autocrine or paracrine manner, consequently leading to tumor initiation and progression (Brown et al., 2021; Endaya et al., 2017). In this study, the human anti-EpCAM sdAbs binding to the EpCAM extracellular domain may inhibit EpCAM cleavage into the extracellular and intracellular domains. In addition, binding of the anti-EpCAM sdAbs to the EpCAM extracellular domain may prevent EpCAM on one cell surface from binding to EpCAM on another cell surface and therefore, decreases homophilic cell-cell adhesion. In addition, it was reported that EpCAM promoted cancer cell proliferation by its intracellular domain generated by TACE and PS-2 (Munz et al., 2009). The anti-EpCAM sdAbs isolated in this study may block the EpCAM cleavage. Studies also showed that EpCAM was used as a novel target for the treatment of leukemia, and anti-EpCAM antibody depleted acute myeloid leukemia (AML) in a mouse model (Zheng et al., 2017). The chemotherapeutic resistance of EpCAM-positive leukemic cells is a consequence of increased WNT5B signaling (Zheng et al., 2017). Our new anti-EpCAM sdAbs may inhibit cancer cell growth by decreasing WNT5B signaling.

A bispecific antibody cotargeting human epidermal growth factor receptor 2 (EGFR2) and type I insulin-like growth factor receptor (IGF-IR) was generated by engineering trastuzumab (anti-EGFR2 antibody) and m590 (anti-IGF-IR antibody) and showed superior anti-tumor activity compared with monospecific antibodies (Chen et al., 2014). Immunotoxin is an antibody-cytotoxin chimeric molecule. In order to optimize the therapeutic efficacy of anti-PD-L1 antibody, an Immunotoxin was constructed by combining an antibody against PD-L1 and a toxin (cucurmosin). The immunotoxin selectively killed PD-L1 positive tumor cells *in vitro* and had good anti-tumor effect on PD-L1 positive human xenograft tumors in nude mice (Zhang et al., 2020). The development of antibody-based therapies has been gradually evolved from a single target to multiple targets. Our human anti-EpCAM sdAbs can also be modified to become bispecific or immunotoxin or by other methods to further increase the therapeutic efficacy.

In summary, an EGF-like repeat epitope located on the EpCAM extracellular domain surface could be chosen as target for sdAb development. The anti-EpCAM sdAbs specifically bound to EpCAM complete extracellular domain and human cancer cells, leading to inhibition of cell proliferation, migration, invasion, and tumor growth *in vivo*. Hence, our study provides compelling evidence that targeting EpCAM for cancer treatment and demonstrates that the anti-EpCAM sdAbs are potential therapeutics for cancer treatment.

## Data Availability

The datasets presented in this study can be found in online repositories. The names of the repository/repositories and accession number(s) can be found below: https://www.ebi.ac.uk/ena, https://www.ebi.ac.uk/ena/data/view/LR535669, https://www.ebi.ac.uk/ena, https://www.ebi.ac.uk/ena/data/view/LR535670, https://www.ebi.ac.uk/ena, https://www.ebi.ac.uk/ena/data/view/LR535671, https://www.ebi.ac.uk/ena, https://www.ebi.ac.uk/ena/data/view/LR535672, https://www.ebi.ac.uk/eva/, https://www.ebi.ac.uk/ena/data/view/LR535673.

## Abbreviations

sdAb, Single-domain antibody; mAb, Monoclonal antibody; EpCAM, Epithelial cell adhesion molecule; CSC, Cancer stem cell; FBS, Fetal bovine serum; PBS, Phosphate-buffered saline; BSA, Bovine serum albumin; ELISA, Enzyme-linked immunosorbent assay; TMB, 3, 3′, 5, 5′-Tetramethylbenzidin; HRP, Horseradish peroxidase; PCR, Polymerase chain reaction; IPTG, Isopropyl-β-d-thiogalactopyranoside; PMSF, Phenylmethylsulfonyl fluoride; DMSO, Dimethyl sulfoxide; Ni-NTA, Nickel nitrilotriacetic acid; SDS-PAGE, Sodium dodecyl sulfate polyacrylamide gel electrophoresis; FITC, Fluorescein isothiocyanate; PE, Phycoerythrin; MTT, 3-(4,5-Dimethylthiazol-2-yl)-2,5 diphenyltetrazolium bromide; DMSO, Dimethyl sulfoxide; OD, Optical density; PI, Propidium iodide; FACS, Flow cytometric analysis; DDP, Cis-platinum; HE, Hematoxylin–eosin; c-caspase-3, Cleaved caspase-3; DAB, Diaminobenzidine; IOD, Integrated optical density; SD, Standard deviation; CDR, Complementarity determining region.
